# Novel Mixed Matrix Membranes Based on Polymer of Intrinsic Microporosity PIM-1 Modified with Metal-Organic Frameworks for Removal of Heavy Metal Ions and Food Dyes by Nanofiltration

**DOI:** 10.3390/membranes12010014

**Published:** 2021-12-23

**Authors:** Anna Kuzminova, Mariia Dmitrenko, Andrey Zolotarev, Aleksandra Korniak, Daria Poloneeva, Artem Selyutin, Alexei Emeline, Alexey Yushkin, Andrew Foster, Peter Budd, Sergey Ermakov

**Affiliations:** 1 St. Petersburg State University, 7/9 Universitetskaya nab., 199034 St. Petersburg, Russia; m.dmitrienko@spbu.ru (M.D.); andrey.zolotarev@spbu.ru (A.Z.); kornyaksandra@gmail.com (A.K.); dashapolon@gmail.com (D.P.); a.selyutin@spbu.ru (A.S.); alexei.emeline@spbu.ru (A.E.); s.ermakov@spbu.ru (S.E.); 2 A. V. Topchiev Institute of Petrochemical Synthesis RAS, 29 Leninsky Prospekt, 119991 Moscow, Russia; halex@ips.ac.ru; 3 Department of Chemistry, University of Manchester, Oxford Road, Manchester M13 9PL, UK; andrew.foster@manchester.ac.uk (A.F.); peter.budd@manchester.ac.uk (P.B.)

**Keywords:** mixed matrix membrane, polymer of intrinsic microporosity, PIM-1, metal-organic frameworks, nanofiltration

## Abstract

Nowadays, nanofiltration is widely used for water treatment due to its advantages, such as energy-saving, sustainability, high efficiency, and compact equipment. In the present work, novel nanofiltration membranes based on the polymer of intrinsic microporosity PIM-1 modified by metal-organic frameworks (MOFs)—MIL-140A and MIL-125—were developed to increase nanofiltration efficiency for the removal of heavy metal ions and dyes. The structural and physicochemical properties of the developed PIM-1 and PIM-1/MOFs membranes were studied by the spectroscopic technique (FTIR), microscopic methods (SEM and AFM), and contact angle measurement. Transport properties of the developed PIM-1 and PIM-1/MOFs membranes were evaluated in the nanofiltration of the model and real mixtures containing food dyes and heavy metal ions. It was found that the introduction of MOFs (MIL-140A and MIL-125) led to an increase in membrane permeability. It was demonstrated that the membranes could be used to remove and concentrate the food dyes and heavy metal ions from model and real mixtures.

## 1. Introduction

Membrane separation methods can be considered as an alternative to traditional separation methods, such as crystallization, sorption, and distillation [1]. Nanofiltration is a pressure-driven membrane separation and concentration method, which is very close to the reverse osmosis process in terms of the separation mechanism and the types of membranes that are used. The intensive development of nanofiltration leads to the use of various polymeric membrane materials. Currently, membranes are being developed based on various polymers, for example: cellulose and its derivatives [2,3], polyamide and its derivatives [4], cellophane [5], polyimide [6], copolymers of 1-trimethylsilyl-1-propyne with 1-(3,3,3-trifluoropropyldimethylsilyl)1-propyne [7], polysulfone [8], polyethersulfone [9], polymer of intrinsic microporosity PIM-1 [10,11], poly [1-(trimethylsilyl)-1-propyne] [11,12,13,14,15], and commercially available membranes [16,17,18,19].

One of the main applications of nanofiltration is as a water treatment from heavy metals. Pure water is a necessary source for the survival of people and other living organisms. The rapid development of technology and industry leads to an increase in water pollution. A large number of heavy metal ions, textile dyes, pharmaceuticals, and pesticides from the energy, fabric, metal, oil, mining, paint and varnish, chemical, and pharmaceutical industries enter the environment every day, leading to water pollutions. Ions of heavy metals, such as copper (Cu^2+^), nickel (Ni^2+^), cobalt (Co^2+^), iron (Fe^3+^), cadmium (Cd^2+^), zinc (Zn^2+^), chromium (Cr^3+^), and lead (Pb^2+^), pose a serious hazard to human health and the environment [20]. Another important and rapidly developing direction is organic solvent nanofiltration (OSN), especially used for the concentration of dyes from organic solvents. The use of nanofiltration for extracting metals and dyes has great advantages over traditional separation methods (sorption, extraction, precipitation), providing effective recovery of target compounds only with a properly selected membrane, and is perspective for various industries, for example in the chemical, textile, petrochemical, and food industries.

The search for membrane material with the tailored properties is still an urgent task for the development of nanofiltration. To solve these set tasks, not only membranes based on pristine polymers are actively used, but also mixed matrix membranes (MMMs). In MMMs, an organic and/or inorganic modifier is added to the polymer matrix, such as polyelectrolytes [21], other polymers [22], carbon nanoparticles [23,24], metal oxides [25,26], and zeolites [27,28], which would change the characteristics of the membranes. In recent years, metal-organic frameworks (MOFs), a new class of crystalline porous materials, have attracted the special attention of researchers. MOFs are porous coordination polymers that are formed by metal ions bonded to polydentate organic molecules (bridging linkers or ligands) in a three-dimensional framework. Metal-organic frameworks are defined as crystalline porous substances, the structure of which is based on strong metal–ligand interactions [29].

Mixed matrix membranes, in which metal-organic frameworks (MOFs) act as fillers, hold much promise due to the simplicity of design and modification of MOFs, as well as the compatibility between MOFs and the polymer matrix [30]. In addition, the introduction of MOFs into a polymer membrane significantly affects the hydrophilic–hydrophobic balance of the surface, sorption characteristics, and free volume of the polymer film due to the porous structure of MOFs. Mixed matrix membranes, where MOFs are a modifier, are used in such membrane processes as nanofiltration [31,32,33], ultrafiltration [34,35,36], pervaporation [37,38], and gas separation [39,40].

MOFs have already been used as modifiers to improve the transport properties of nanofiltration membranes based on polyamide [41], polyethersulfone [42,43], cellulose acetate [42], polyvinylidene fluoride [42], polyethyleneimine/trimesic acid [31], polyimide [44], polyethyleneimine [32,45], polyvinyl alcohol [33], and polyphenylene sulfone [46]. Despite the fact that MOFs are promising modifiers for creating nanofiltration mixed matrix membranes, there are few works in the literature in this direction. It is also worth noting that there are no reported data in the literature on the modification of a polymer of intrinsic microporosity with MOFs for nanofiltration. In this research, MIL-125 and MIL-140A were chosen as modifiers because they contain necessary functional groups (-NH_2_ and -COOH) with which the interaction with PIM-1 could be reached that caused the changing of the physicochemical and transport characteristics of the nanofiltration membrane [47]. Moreover, these MOFs have unique properties, such as high stability with respect to water and organic solvents, an extremely high thermal stability, and a stable and size-appropriate window for the nanofiltration membrane. MIL-125 and/or its derivatives (NH_2_-MIL-125) were used to modify mixed matrix membranes for gas separation [47,48,49], pervaporation [50], membrane distillation [51], and nanofiltration [31]. Data on the incorporation of MIL-125 and MIL-140A into polymers of intrinsic microporosity PIM-1 to create nanofiltration membranes are not presented in the literature.

PIM-1 is a condensation product of 5,5′,6,6′-tetrahydroxy-3,3,3′,3′-tetramethyl-1,1′-spirobisindane and tetrafluoroterephthalonitrile. PIM-1 is a hydrophobic polymer with polar groups, and it has a large internal surface area of 772 m^2^/g [11]. Membranes based on PIM-1 have a microporous structure with internal membrane cavities of ~1 nm in size, which are formed naturally during the formation of a polymer film due to the rigid structure of macromolecular chains [52]. PIM-1 polymer is used in membranes for gas separation [53,54], pervaporation [55,56], and nanofiltration [52,57]. Zn(pyrz)_2_(SiF_6_) [58], UiO-66 [59], MOF-801 [53], MIL-101 [60], crystals of silicalite-1 [61], and graphene oxide and its derivatives [62] have been used as modifiers to create mixed matrix membranes based on PIM-1, but most of the mixed matrix membranes are used in the process of gas separation rather than nanofiltration.

The aim of the present work was to develop novel nanofiltration membranes based on a polymer of intrinsic microporosity PIM-1 modified by metal-organic frameworks (MOFs)—MIL-140A and MIL-125—for the removal of heavy metals and dyes. We were able to improve the transport properties of the developed PIM-1 and PIM-1/MOFs membranes through modification, due to the following properties of the used MOFs: porous structure of MOFs, stability in water and organic solvent, hydrophobic/hydrophilic properties, and different crystal structure. All these factors affect the free volume and surface of the developed membranes. The structural and physicochemical properties of the developed PIM-1 and PIM-1/MOFs membranes were studied by Fourier transform infrared spectroscopy (FTIR), microscopic methods (SEM and AFM), and contact angle measurement. Transport properties of the developed PIM-1 and PIM-1/MOFs membranes were tested in the nanofiltration of model and real mixtures containing food dyes and heavy metal ions.

## 2. Materials and Methods

In this work, at least three samples of each type of supported membranes were prepared to study the physicochemical and transport properties. All investigations for these membrane series were carried out under the same conditions at ambient temperature to evaluate the effect of membrane modification and to avoid property differences from variation or non-variation in the experimental or analytical conditions. For the accuracy of all characteristics, data were collected in triplicate and the average values were used for subsequent presentation.

### 2.1. Materials

Polymer of intrinsic microporosity, PIM-1 (*M_w_* = 201,300, Đ = 2.1), was synthesized based on the method previously reported in the literature [63] and used as a membrane material. Metal-organic frameworks MIL-125 (specific surface area 1565 m^2^/g, average pore width 1.12 nm) and MIL-140A (specific surface area 493 m^2^/g, average pore width 0.87 nm) were used as polymer modifiers and provided by the research group “Photoactive nanocomposite materials” of St. Petersburg State University (St. Petersburg, Russia). Chloroform was used as a solvent without additional treatment and purchased from “Vekton” (St. Petersburg, Russia). Ultrafiltration membrane UPM-20^®^ was purchased from ZAO STC “Vladipor” (Vladimir, Russia) and used as support to obtain supported membranes. CuSO_4_, Cu(NO_3_)_2_, Pb(NO_3_)_2_, Cd(NO_3_)_2_, and yellow “sunset” dye were purchased from “NevaReactive” (St. Petersburg, Russia). Alfazurin was purchased from Sigma–Aldrich (St. Petersburg, Russia).

### 2.2. Membrane Preparation

PIM-1 (4 wt.%) solution was prepared by dissolving the PIM-1 powder in chloroform with stirring for 5 h. PIM-1/MOFs composites were prepared by the solid-phase method as follows: MOFs were ground in an agate mortar, then PIM-1 powder was added. The resulting mixture of PIM-1 and MOF was dissolved in chloroform with stirring for 5 h. The resulting suspensions were further sonicated for more than 30 min at room temperature for degassing of the polymer solution, and 5–25 wt.% MOFs (MIL-125 and MIL-140A) with respect to the polymer weight were introduced into the PIM-1 matrix. PIM-1 membranes modified with 20 wt.% MOFs (MIL-125 and MIL-140A) demonstrated optimal transport properties (the trade-off high permeability and high rejection coefficient). The increase of MOFs to 25 wt.% in the PIM-1 matrix did not lead to irreproducibility of transport characteristics due to uneven agglomeration of the modifier particles in the membrane. Supported membranes were developed by applying a thin layer of polymer or composite on a porous UPM-20 substrate by the physical adsorption method, as follows: the UPM-20 substrate was fixed on a hollow ring, the polymer suspension was casted from the ring side onto the substrate, the excess of the casting solution was removed, and the membrane was covered with a glass lid of a Petri dish. This drying method provides gradual solvent evaporation and limits the possibility of pore holes in the membrane selective layer [64]. The thickness of the selective layer of the developed supported membranes was varied from 500 nm to 5 μm. It was found that the supported PIM and PIM-1/MOFs membranes with the thickness of 5 ± 0.2 µm of the selective layer demonstrated optimal transport characteristics: the trade-off of the permeability, the rejection coefficient, and the concentration coefficient. The selective layer thickness of supported membranes was controlled and measured by scanning electron microscopy (SEM).

### 2.3. Preparation of Samples of Model Mixture for Analysis

#### 2.3.1. Solutions of Heavy Metal Ions in Water

CuSO_4_ solution containing copper ions (Cu^2+^) in water was prepared by dissolving the salt in water with stirring for 60 min at ambient temperature. The initial concentration of copper ions in water was 100 mg/L. For the preparation of a solution with a mixture of heavy metal ions, nitrates of heavy metal ions (Cu^2+^, Pb^2+^, Cd^2+^) were chosen as they were all soluble salts. An electrolyte solution containing Cu^2+^, Pb^2+^, and Cd^2+^ ions was prepared by dissolving Cu(NO_3_)_2_, Pb(NO_3_)_2_, and Cd(NO_3_)_2_ salts in water with constant stirring for 3 h at ambient temperature. The concentration of metal ions was 50 mg/L in each case.

#### 2.3.2. Solutions of Food Dyes in Ethanol

Food dye solutions were prepared by dissolving a weighed portion of the dye (alfazurin (E133) or yellow “sunset” (E110)) in ethanol with stirring for 60 min at ambient temperature. The initial concentration of the dye was 10 mg/L.

### 2.4. Preparation of Samples of Real Mixture for Analysis

#### 2.4.1. Untreated Wastewater Samples from Galvanic Production

A sample of untreated wastewater was provided by LLC “Galvanik” (St. Petersburg, Russia), which contained Cd^2+^, Cr^3+^, Cu^2+^, Fe^3+^, Ni^2+^, and Zn^2+^ ions. For nanofiltration, untreated wastewater was also diluted 1000 and 100 times by distilled water at ambient temperature. The resulting water sample was filtered through a “blue ribbon” filter prior to dilution and analysis.

#### 2.4.2. Caramel Samples Containing Dyes

The commercially available “Raskras-ka” caramel manufactured by Chaozhou Tiaoxiang Foodstuff Factory was dissolved in a water/ethanol mixture (50/50 vol.%) for the analysis of food dyes (E110, E129, and E133). Thirty pieces of caramel of each color were dissolved in water at 50 °C and then diluted with ethanol at ambient temperature, and then the caramel solution was filtered through a “blue ribbon” filter.

### 2.5. Scanning Electron Microscopy

Microphotographs of the cross-section and surface of supported PIM-1 and PIM-1/MOFs membranes were obtained by scanning electron microscopy (SEM) with a Zeiss Merlin SEM microscope. To obtain a cross-section of the membrane, the samples were submerged in liquid nitrogen and fractured perpendicular to the surface, and then attached to carbon tape. To obtain the surface SEM micrographs, a piece of membrane 2 × 2 mm in size was attached to carbon tape. The morphology of the samples was studied using secondary electrons at a voltage of 1 kV.

### 2.6. Infrared Spectroscopy

The BRUKER-TENSOR 27 spectrometer was used to determine the structural changes in the supported PIM-1 and PIM-1/MOFs membranes. IR spectroscopy was performed in the range 600–4000 cm^−1^ at 25 °C. The measurement of the membranes was carried out from the side of the selective layer of the supported membrane using an attenuated total reflectance (ATR) accessory (PIKE Technologies, Moscow, Russia).

### 2.7. Atomic Force Microscopy

The atomic force microscope NT-MDT NTegra Maximus was used to study the surface topography of supported PIM-1 and PIM-1/MOFs membranes. To obtain the AFM images, a piece of membrane 5 × 5 mm in size was measured by tapping mode.

### 2.8. Investigation of Contact Angles

To study the surface characteristics of supported PIM-1 and PIM-1/MOFs membranes, the contact angles of water were measured from the side of the selective layer by the sessile drop method at ambient temperature on a Goniometer LK device (NPK Open Science, Krasnogorsk, Russia). A drop of 4 μL was applied to the surface of the selective layer using a chromatographic syringe. The calculation of the obtained results was carried out in the “DropShape” program.

### 2.9. Nanofiltration Experiment

**Figure 1:**
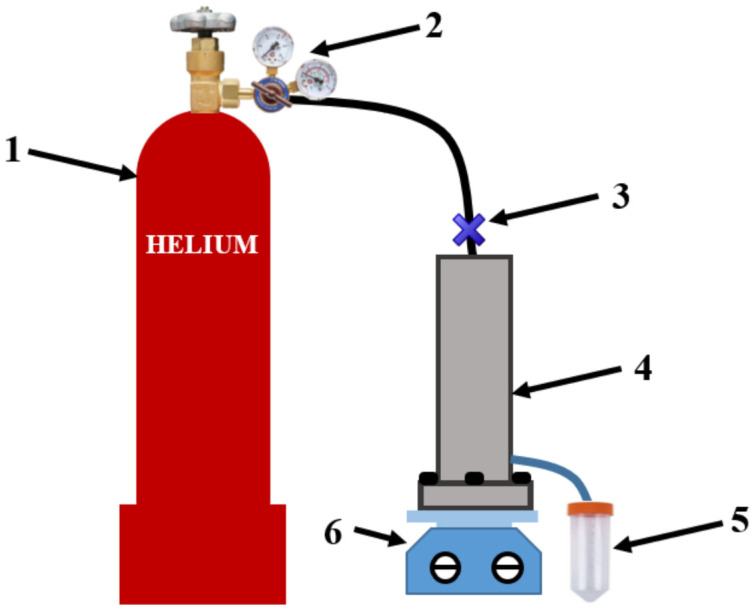
The scheme of the nanofiltration setup: 1—helium cylinder, 2—reducer, 3—gas supply opening/closing valve, 4—nanofiltration cell, 5—test tube with permeate, 6—magnetic stirrer.

To study the transport properties of the developed nanofiltration membranes, a nanofiltration laboratory dead-end cell with an effective membrane area of 10 × 10^−4^ m^2^ was used in stationary mode with pressure applied to the membrane up to 50 atm at 22 °C with stirring (Figure 1). For dyes and caramel solutions, the feed volume was 400 mL, while the volume of aqueous solutions with metal ions was 300 mL, since the membrane water permeability was lower. After the nanofiltration experiment, the NF cell was washed with ethanol and water. After nanofiltration of a real object containing heavy metal ions, the cell was washed with a Trilon B solution in water (5 g/L), and then with water. The collection of permeate was carried out during different time periods for various feed mixtures to obtain the minimum volume of 5 mL of permeate. In the case of nanofiltration of a real mixture, the collection of permeate could take up to 48 h. It should be noted that for a series of each membrane type, the permeate was collected for the same time periods to ensure the reproducibility of the transport parameters and to conduct the nanofiltration experiment under the same conditions.

The permeation flux, *J* (kg/(m^2^h)), of a membrane is described as the amount of liquid passing through a unit of membrane area per unit of time. The parameter is calculated according to the described equation [65]:

(1) $$J=\frac{m}{A\cdot t},$$

where *m* is the mass of permeate (kg) formed during time *t* (h) from the membrane surface with area *A* (m^2^).

Permeability, *L* ((kg·m)/(m^2^·h·atm)), of a membrane is calculated by the equation [66]:

(2) $$L=\frac{J\cdot l}{\Delta P}=\frac{m\cdot l}{A\cdot t\cdot \Delta P},$$

where *m* is the mass of permeate (kg), *t* (h) is time, *A* (m^2^) is membrane surface area, *l* (m) is the thickness of the selective layer, and ∆*P* (atm) is pressure.

The rejection coefficient of the component is calculated by the equation:

(3) $$R=\left(1-\frac{C_{perm}}{C_{feed}}\right)\cdot 100\%,$$

where C*_perm_* and C*_feed_* are the concentration of the component in the permeate and the feed, respectively.

The concentration coefficients were also calculated in this work, which showed the possibility of concentrating components from the initial model or real mixtures. The concentration process was carried out in the process of nanofiltration, in which a different amount of permeate passed over the same time with the different concentration of the components in the permeate depending on the membrane and feed. The concentration coefficient is calculated by the equation:

(4) $$K=\frac{C_{conc}}{C_{feed}}\cdot 100\%,$$

where C*_conc_* and C*_feed_* are the concentration of the component in the concentrate and the feed, respectively.

For the accuracy of all transport characteristics, data were collected in triplicate and the average was used for subsequent presentation.

### 2.10. Spectrophotometric Analysis

The concentration of food dyes in the model mixture in ethanol was studied using a PE-5400UF spectrophotometer (“ECROSKHIM”, St. Petersburg, Russia) at wavelengths of 628 nm for Alfazurin (E133) and 483 nm for yellow “sunset” (E110). The dye spectra were recorded using glass cells with an optical path length of 1 cm.

**Figure 2:**
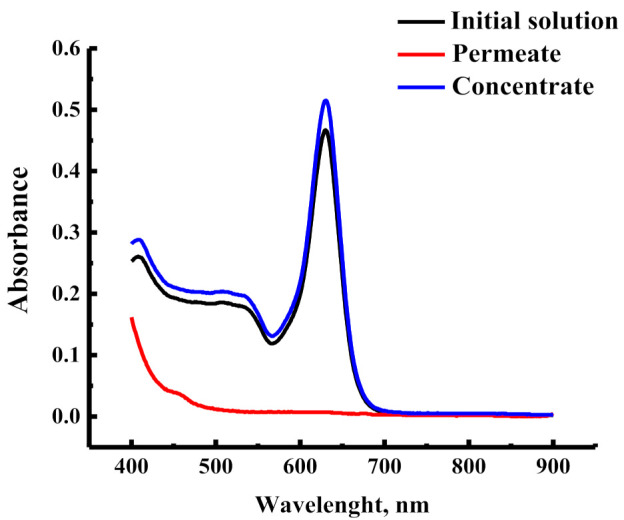
The spectra of the initial solution of caramel, permeate, and concentrate for the PIM-1 membrane.

The concentration of food dyes in the caramel solution was studied using an SF-102 spectrophotometer (“Akvilon SZ”, St. Petersburg, Russia) at a wavelength range of 400–900 nm. Figure 2 shows the spectrum of the initial solution of caramel, permeate, and concentrate for the PIM-1 membrane. The dye spectra were recorded using glass cells with an optical path length of 1 cm.

### 2.11. Stripping Voltammetry

The concentration of metal ions (Cu^2+^, Pb^2+^, Cd^2+^) in the model solution was studied by stripping voltammetry on a TA-4 voltammetric analyzer: silver chloride electrodes were used as a reference and auxiliary electrodes; a mercury film electrode was used as working electrode. Then, 10 mL of water, 0.2 mL of formic acid, and 0.04 mL of permeate were added to the measuring cups. Simultaneously, 3 parallel measurements were carried out with one sample of permeate.

### 2.12. Inductively Coupled Plasma Atomic Emission Spectrometry

The concentration of metal ions in a real mixture was studied by atomic emission spectrometry on an ICPE-9000 optical emission spectrometer (Shimadzu, Japan) at ambient temperature. Standard samples of the analyzed elements for calibration solutions were prepared from the MERCK multielement standard in 0.1N HNO_3_. The calibration solution range was: 0.001–100 mg/dm^3^. The wavelengths for metal ions were as follows: Cd^2+^ 228.802, Cr^3+^ 205.552, Cu^2+^ 327.396, Fe^3+^ 259.940, Ni^2+^ 231.604, and Zn^2+^ 206.200 nm.

## 3. Results

### 3.1. Transport Properties of Nanofiltration Membranes

#### 3.1.1. Solvent Permeability

**Figure 3:**
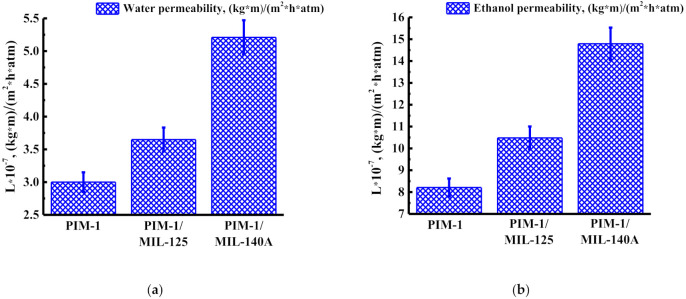
Permeability of membranes during nanofiltration of (**a**) water and (**b**) ethanol at 50 atm.

The transport properties of nanofiltration membranes were studied in the nanofiltration of water and ethanol at 50 atm at ambient temperature, and the permeability of water and ethanol for membranes is shown in Figure 3.

The data presented in Figure 3 demonstrate that the membrane water permeability is less than the membrane ethanol permeability for all nanofiltration membranes. The introduction of MIL-125 leads to an increase in the membrane water permeability of ~21% and an increase in the membrane ethanol permeability of ~28%, compared to the unmodified PIM-1 membrane. The introduction of MIL-140A leads to an increase in the membrane water permeability of ~73% and an increase in the membrane ethanol permeability of ~80% compared to the unmodified PIM-1 membrane. The increase in permeability in the case of MIL-125 introduction may be associated with an increase in the size of the “pores” on the membrane surface (confirmed by SEM in the Section 3.2.2) and an increase in roughness (confirmed by AFM in the Section 3.2.3). The increase in permeability in the case of the introduction of MIL-140A may be associated with an increase in roughness (confirmed by the AFM in the Section 3.2.3) and a decrease in the contact angle of water, that is, hydrophilization of the surface (data from the Section 3.2.4).

#### 3.1.2. Concentration of Heavy Metal Ions

**Figure 4:**
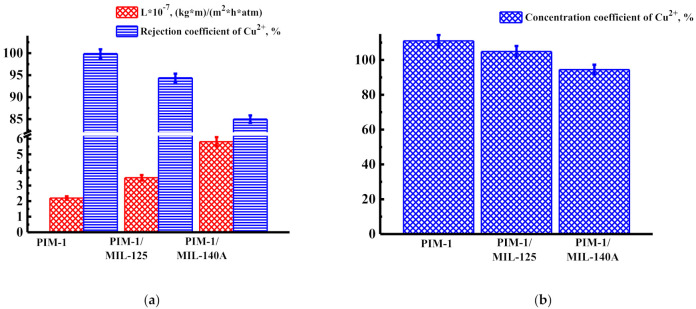
Transport properties: (**a**) permeability and rejection coefficient and (**b**) concentration coefficient during nanofiltration of the solution containing copper ions (Cu^2+^) at 50 atm.

The transport properties of nanofiltration membranes were also studied in the nanofiltration of solutions containing copper ions (Cu^2+^) and a mixture of heavy metal ions (Cd^2+^, Pb^2+^, Cu^2+^) in water at ambient temperature. After the experiment, the cells were washed with water. Figure 4 shows the permeability, rejection coefficient, and concentration coefficient at 50 atm for unmodified and modified membranes during nanofiltration of solution with copper ions.

The data presented in Figure 4a show that the introduction of MIL-125 and MIL-140A leads to an increase in permeability, but to a decrease in the rejection coefficients of copper ions. The membrane modified with MIL-140A (PIM-1/MIL-140A membrane) had the highest permeability value—5.81 × 10^−7^ (kg·m)/(m^2^·h·atm)—during nanofiltration of the solution containing copper ions. The data presented in Figure 4b demonstrate that all the nanofiltration membranes are promising for the concentration of heavy metal ions (Cu^2+^) from individual solutions because values of the concentration coefficients of Cu^2+^ are high (>94%).

**Figure 5:**
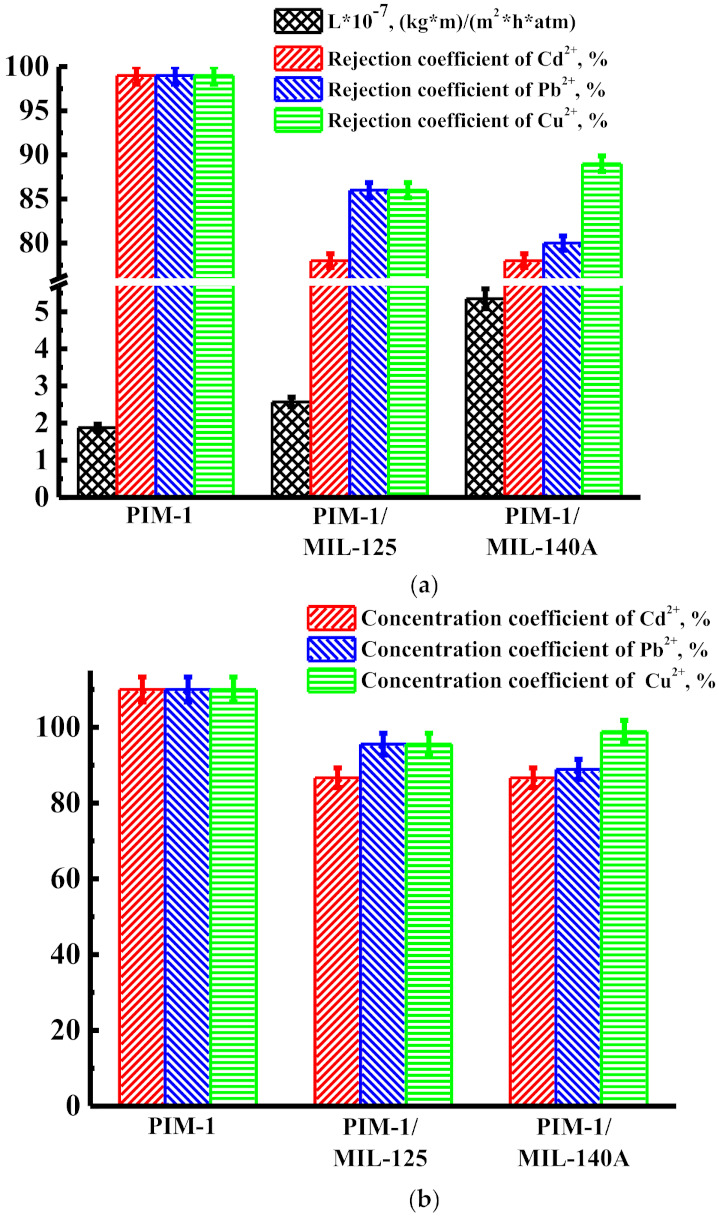
Transport properties: (**a**) permeability and rejection coefficients and (**b**) concentration coefficients during nanofiltration of the solution containing a mixture of heavy metal ions (Cd^2+^, Pb^2+^, Cu^2+^) at 50 atm.

Figure 5 shows the permeability, rejection coefficient, and concentration coefficient at 50 atm for unmodified and modified membranes during nanofiltration of solution with a mixture of heavy metal ions (Cd^2+^, Pb^2+^, Cu^2+^).

The data presented in Figure 5a show that the introduction of MIL-125 and MIL-140A leads to an increase in permeability, but to a small decrease in the rejection coefficients (up to 78%) of ions of all studied heavy metals. The membrane modified with MIL-140A (PIM-1/MIL-140A membrane) had the highest permeability value—5.35 × 10^−7^ (kg·m)/(m^2^·h·atm)—during nanofiltration of the solution containing a mixture of heavy metal ions. In addition to permeability, the concentration coefficient also depends on the rejection coefficient. The data presented in Figure 5b demonstrate that all the nanofiltration membranes are promising for the concentration of heavy metal ions from a mixture of heavy metal ions, and all values of the concentration coefficients are high (>86%).

**Figure 6:**
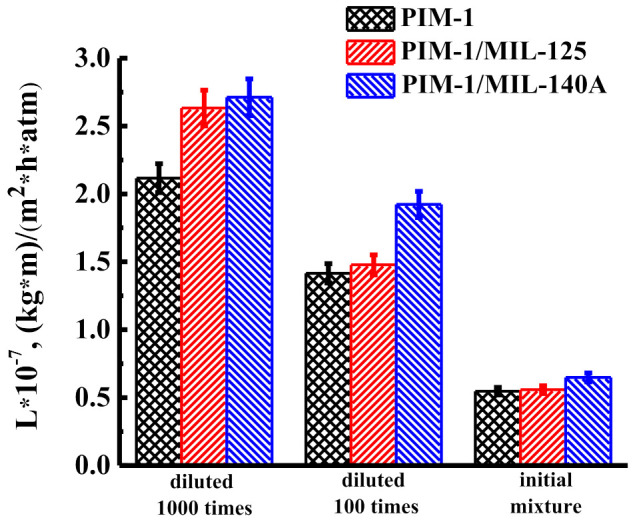
Permeability of PIM-1, PIM-1/MIL-125, and PIM-1/MIL-140A membranes during the nanofiltration at 50 atm: a sample of untreated wastewater (initial mixture), a sample of untreated wastewater, diluted 100 times, and a sample of untreated wastewater diluted 1000 times.

**Figure 7:**
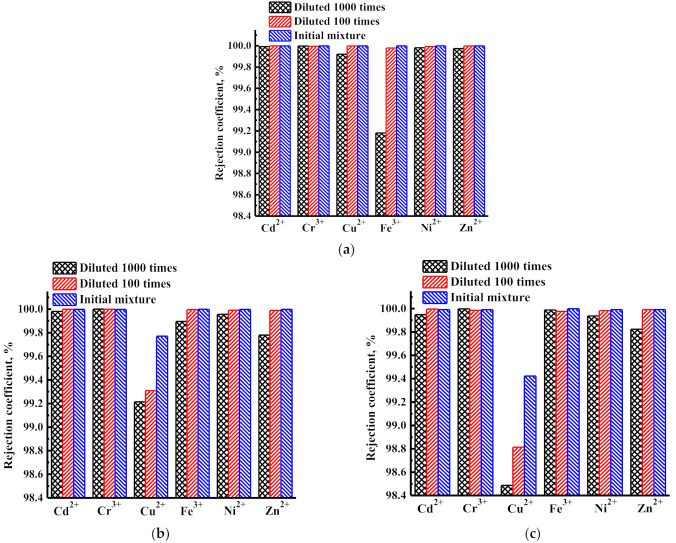
Rejection coefficients of heavy metal ions for (**a**) PIM-1, (**b**) PIM-1/MIL-125, and (**c**) PIM-1/MIL-140A membranes during the nanofiltration at 50 atm: a sample of untreated wastewater (initial mixture), a sample of untreated wastewater, diluted 100 times, and a sample of untreated wastewater diluted 1000 times.

**Figure 8:**
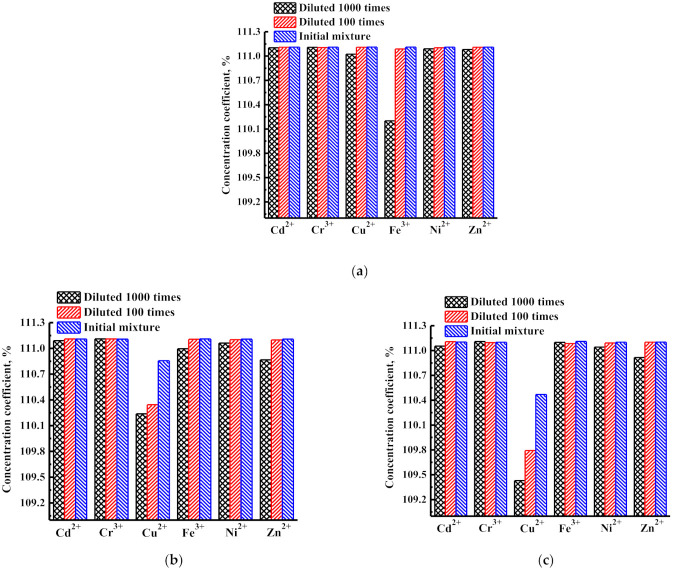
Concentration coefficients of heavy metal ions for (**a**) PIM-1, (**b**) PIM-1/MIL-125, and (**c**) PIM-1/MIL-140A membranes during the nanofiltration at 50 atm: a sample of untreated wastewater (initial mixture), a sample of untreated wastewater, diluted 100 times, and a sample of untreated wastewater diluted 1000 times.

The developed nanofiltration PIM-1 and PIM-1/MOFs membranes were used to concentrate heavy metals from untreated wastewater provided by LLC “Galvanik”. The separation results are shown in Figure 6, Figure 7 and Figure 8. The experiment was carried out at 50 atm at ambient temperature. After the experiment, the cells were washed with water.

Figure 6 shows the permeability of the developed membranes in the nanofiltration of several solutions at 50 atm: a sample of untreated wastewater, a sample of untreated wastewater, diluted 100 times, as well as a sample of untreated wastewater diluted 1000 times.

The data presented in Figure 6 show that with an increase in the concentration of heavy metals in untreated wastewater for nanofiltration membranes, a decrease in permeability is observed: from 2.12 × 10^−7^ to 0.55 × 10^−7^ (kg·m)/(m^2^·h·atm) for PIM-1, from 2.63 × 10^−7^ to 0.56 × 10^−7^ (kg·m)/(m^2^·h·atm) for PIM-1/MIL-125, and from 2.71 × 10^−7^ to 0.65 × 10^−7^ (kg·m)/(m^2^·h·atm) for PIM-1/MIL-140A membranes. It should be noted that the PIM-1/MIL-140A membrane has the highest permeability values, as in the case with model solutions of a mixture of heavy metal ions (Figure 5).

Based on the data obtained from nanofiltration experiments with untreated wastewater containing heavy metals, rejection and concentration coefficients of heavy metal ions were calculated and presented in Figure 7 and Figure 8, respectively. Rejection coefficients of heavy metal ions (Cd^2+^, Cr^3+^, Cu^2+^, Fe^3+^, Ni^2+^, Zn^2+^) for PIM-1 and PIM-1/MOFs membranes are shown in Figure 7.

High rejection coefficients were obtained for all heavy metal ions from aqueous solutions (Cd^2+^, Cr^3+^, Cu^2+^, Fe^3+^, Ni^2+^, Zn^2+^) for the studied nanofiltration PIM-1, PIM-1/MIL-125, and PIM-1/MIL-140A membranes. The smallest rejection coefficients were noted for copper ions (Cu^2+^) in modified membranes (for the PIM-1/MIL-125 membrane, they were higher than for the PIM-1/MIL-140A membrane).

The values of the concentration coefficients of heavy metal ions (Cd^2+^, Cr^3+^, Cu^2+^, Fe^3+^, Ni^2+^, Zn^2+^) for PIM-1 and PIM-1/MOFs membranes are shown in Figure 8.

High concentration coefficients (more than 100%) were obtained for all metal ions for supported nanofiltration PIM-1, PIM-1/MIL-125, and PIM-1/MIL-140A membranes.

The differences in the rejection coefficients, permeability, and concentration coefficients are associated, of course, with two factors. First, the PIM-1/MIL-140A membrane has a less dense packing of polymer and modifying particles in the composite structure (SEM data in the Section 3.2.2), while MIL-125 is embedded in the structure and “pores” of PIM-1 in PIM-1/MIL-125 membranes. Second, the dynamic sorption characteristics for the studied metal ions are different. It is known that the dynamic capacity of many sorbents for the copper ion is lower since the sorption equilibrium is established faster [67,68,69,70,71]. A decrease in the rejection and concentration coefficients for copper ion using modified membranes can be associated with the characteristics of the copper ion as a Jahn-Teller ion—the formation of labile complexes with various donor atoms and a distorted structure of the resulting environment, which is not observed for cadmium, chromium, iron, nickel, and zinc ions.

#### 3.1.3. Concentration of Food Dyes

The transport properties of nanofiltration membranes were also studied in the nanofiltration of anionic food dye solutions in ethanol at 40 atm at ambient temperature. After the experiment, the cells were washed with ethanol and water.

**Figure 9:**
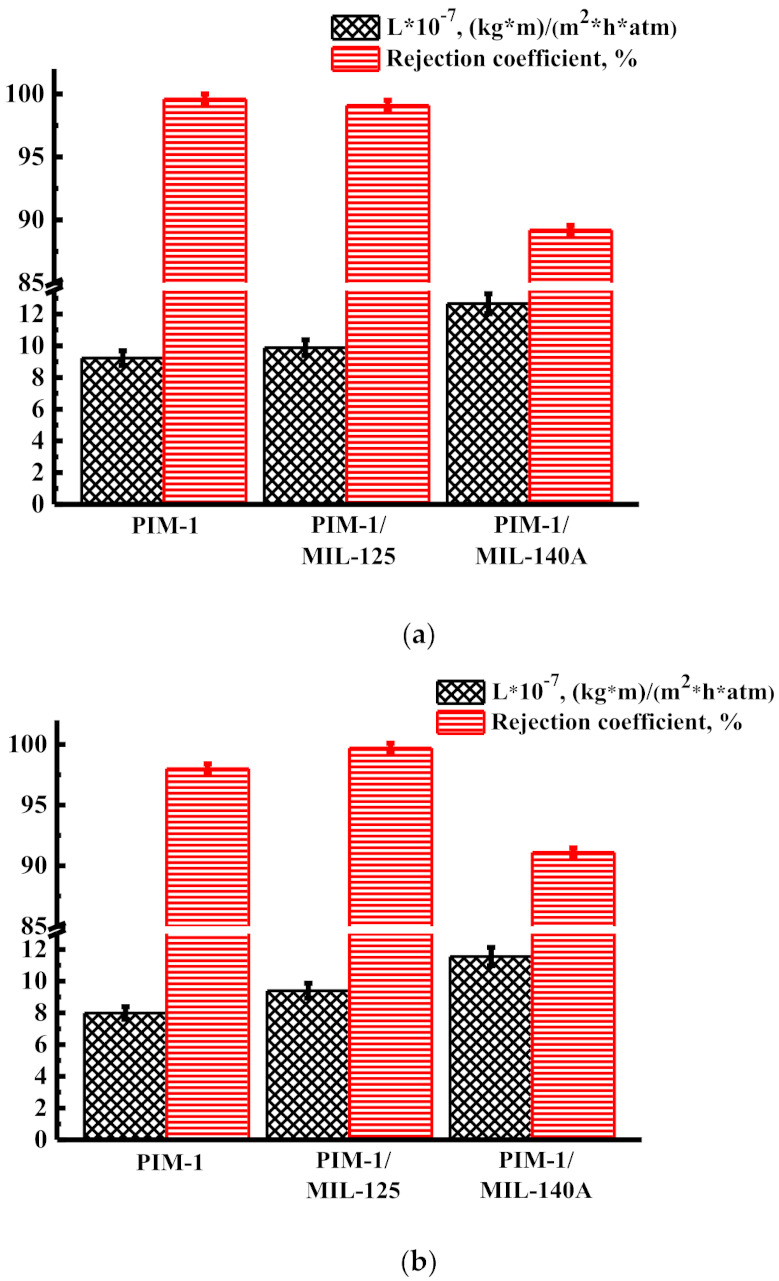
Transport properties of membranes (permeability and rejection coefficient) during nanofiltration of (**a**) alfazurin (E133) and (**b**) yellow “sunset” (E129) dye solutions in ethanol at 40 atm.

Figure 9 shows the permeability and rejection coefficient for unmodified and modified membranes during nanofiltration of solutions of alfazurin (E133) (Figure 9a) and yellow “sunset” dye (E110) (Figure 9b) in ethanol.

The data presented in Figure 9 demonstrate that the introduction of MIL-125 leads to an increase in permeability of ~7% during nanofiltration of a solution of alfazurin in ethanol and of ~17% during nanofiltration of a yellow “sunset” dye solution in ethanol, compared with the unmodified PIM-1 membrane maintaining a high level of rejection coefficient of dyes (more than 99% for both anionic food dyes). The introduction of MIL-140A leads to an increase in permeability of ~37% during nanofiltration of a solution of alfazurin in ethanol and of ~44% during nanofiltration of a solution of the yellow “sunset” dye in ethanol compared to an unmodified PIM-1 membrane maintaining a high rejection coefficient (89% and 91% for alfazurin and yellow “sunset” solutions, respectively).

**Figure 10:**
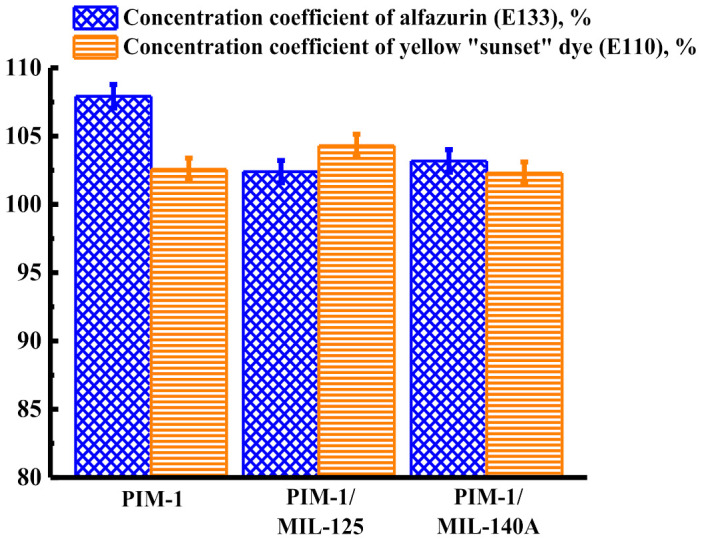
Concentration coefficient of alfazurin (E133) and yellow “sunset” dye (E129) solutions at 40 atm for unmodified and modified membranes.

Figure 10 shows the concentration coefficient of alfazurin (E133) and yellow “sunset” (E110) dye from ethanol solutions at 40 atm for unmodified and modified membranes. The concentration of dyes in nanofiltration occurs due to the rejection of dyes by the membrane, while the solvent passes through the membrane.

The data presented in Figure 10 demonstrate that all developed nanofiltration membranes can be used to concentrate the dyes alfazurin (E133) and yellow “sunset” (E110) from ethanol solutions since all concentration coefficients are >100%.

**Figure 11:**
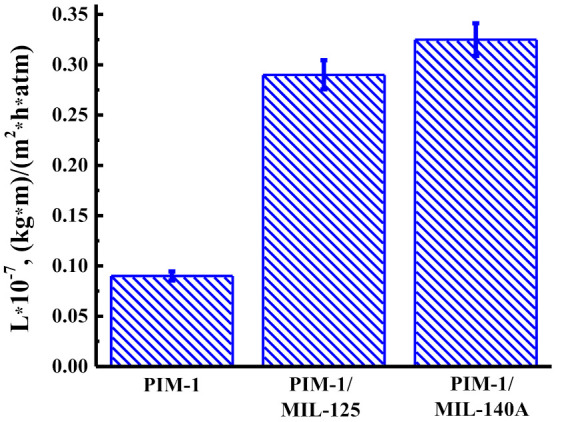
The permeability during nanofiltration of dissolved caramel in the water/ethanol mixture (50/50 vol.%) for nanofiltration PIM-1, PIM-1/MIL-125, and PIM-1/MIL-140A membranes at 50 atm.

**Figure 12:**
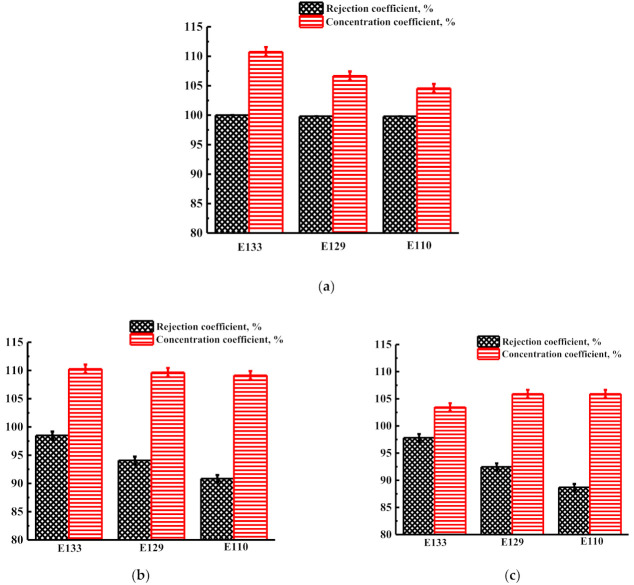
The rejection and concentration coefficients of food dyes (E110, E129, and E133) for (**a**) PIM-1, (**b**) PIM-1/MIL-125, and (**c**) PIM-1/MIL-140A membranes during the nanofiltration of dissolved caramel in a mixture of water/ethanol (50/50 vol.%) at 50 atm.

The characteristics of nanofiltration PIM-1 and PIM-1/MOFs membranes were studied in the concentration of food dyes (E110, E129, and E133) from the commercially available caramel brand “Raskras-ka”, the manufacturer of which is Chaozhou Tiaoxiang Foodstuff Factory. The permeability of the membranes was studied (Figure 11) and the rejection and concentration coefficients of food dyes were calculated (Figure 12).

Figure 11 shows the permeability for nanofiltration of dissolved caramel in a water/ethanol mixture (50/50 vol.%) for nanofiltration PIM-1, PIM-1/MIL-125, and PIM-1/MIL-140A membranes at 50 atm at ambient temperature. After the experiment, the cells were washed with water.

It was found that the introduction of modifiers into the PIM-1 matrix led to an increase in the permeability of the dissolved caramel in the water/ethanol mixture (50/50 vol.%). The highest permeability values were noted for the PIM-1/MIL-140A membrane.

Figure 12 shows the values of the rejection and concentration coefficients of food dyes (E110, E129, and E133) for PIM-1 and PIM-1/MOFs membranes during the nanofiltration of dissolved caramel in a mixture of water/ethanol (50/50 vol.%) at 50 atm.

It was found that the rejection coefficient of E133 food dye was higher than E129 and E110 food dyes by modified nanofiltration PIM-1/MIL-125 and PIM-1/MIL-140A membranes. For the unmodified PIM-1 membrane, high rejection coefficients were noted for all three food dyes. The concentration coefficients of food dyes of all three membranes, PIM-1, PIM-1/MIL-125, and PIM-1/MIL-140A, were more than 100%.

Thus, the introduction of MIL-125 and MIL-140A into the PIM-1 matrix leads to an increase in membrane permeability in the nanofiltration of model and real mixtures, maintaining a high level of rejection and concentration coefficients.

### 3.2. Structure and Physicochemical Properties Investigation of Nanofiltration Membranes

#### 3.2.1. Study by Fourier Transform Infrared Spectroscopy (FTIR)

**Figure 13:**
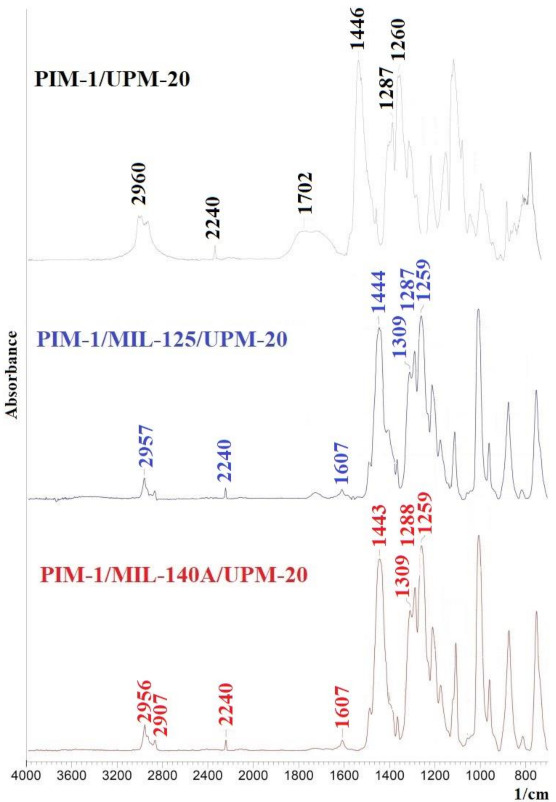
IR spectra of nanofiltration-supported PIM-1 and PIM-1/MOFs membranes.

The structural characteristics of nanofiltration-supported PIM-1 and PIM-1/MOFs membranes were studied using IR spectroscopy. IR spectra are measured from the side of the selective layer at ambient temperature and shown in Figure 13.

The characteristic peaks are presented in the IR spectra of PIM-1. The peak at 2240 cm^−1^ indicates the stretching of the nitrile group, the peak at 2800–2900 cm^−1^ corresponds to –C–H [72], the peak at 1702 cm^−1^ corresponds to C=N [73], the peak at 1446 cm^– 1^ corresponds to –CH bonds from the C-CH_3_ and -CH_2_ groups [53], and the group of peaks at 1250–1350 cm^−1^ corresponds to the stretching of the -C-O- bond [72]. The introduction of MOFs into the PIM-1 matrix leads to small changes in the IR spectra: insignificant displacements of the peaks and changes in their intensity occur, which may indicate an overlapping of the characteristic MOFs peaks with the peaks for PIM-1. Additionally, in the spectra of the modified PIM-1 membranes, there is a peak at ~1607 cm^−1^, which corresponds to aromatic C=C bonds [53].

#### 3.2.2. Study by Scanning Electron Microscopy (SEM)

**Figure 14:**
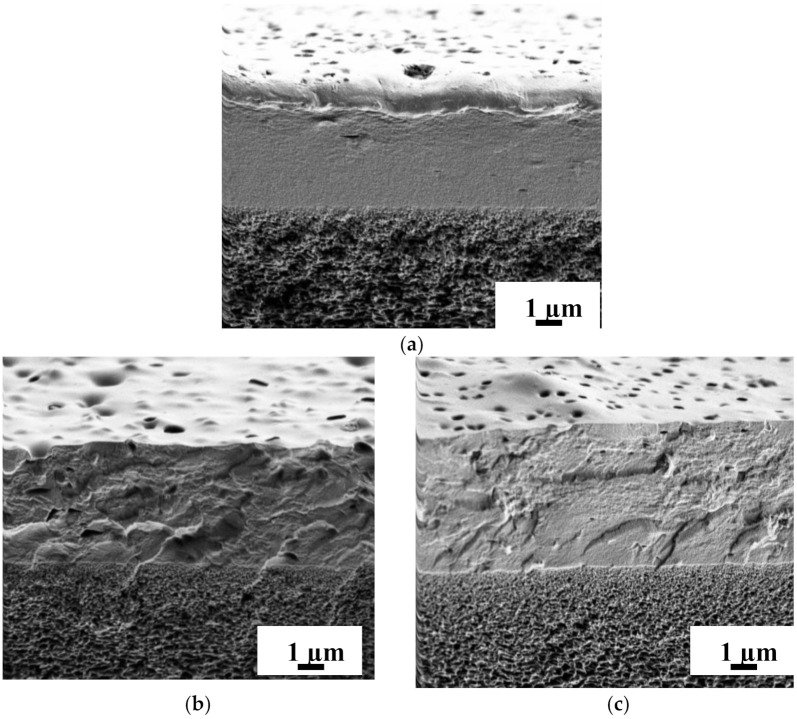
SEM micrographs of a cross-section of nanofiltration-supported (**a**) PIM-1, (**b**) PIM-1/MIL-125, and (**c**) PIM-1/MIL-140A membranes.

**Figure 15:**
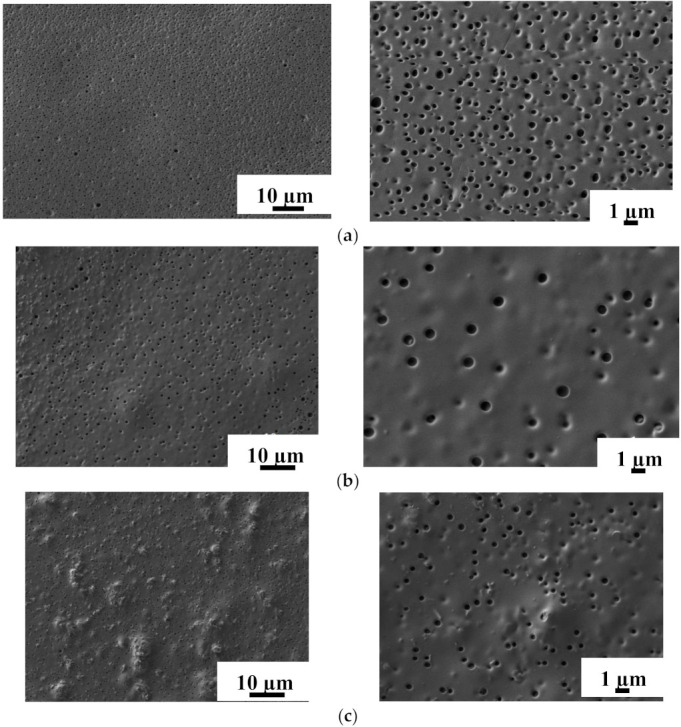
SEM micrographs of the surface at different magnifications of nanofiltration-supported (**a**) PIM-1, (**b**) PIM-1/MIL-125, and (**c**) PIM-1/MIL-140A membranes.

The internal structure and surface morphology of nanofiltration-supported PIM-1 and PIM-1/MOFs membranes were studied by SEM. SEM micrographs of the cross-section and surface for nanofiltration-supported PIM-1 and PIM-1/MOFs membranes are shown in Figure 14 and Figure 15, respectively.

According to cross-sectional SEM micrographs (Figure 14), the thickness of the selective layer of the supported membranes was uniform and equal to 5 ± 0.2 μm. The small variation of 0.2 μm did not affect the reproducibility of the transport properties of membranes, since every series of each membrane (at least 3 samples) was studied in nanofiltration to confirm the constancy of transport properties. The SEM micrograph of the unmodified membrane (Figure 14a) shows a smooth structure of the cross-section. The introduction of MIL-125 and MIL-140A into the PIM-1 matrix results in a coarser cross-section structure: irregularities, “cavities”, and “bulges” associated with the presence of MOFs.

The SEM micrographs shown in Figure 15 indicate the presence of “pores” on the surface that are not hollow according to the cross-section SEM micrographs (Figure 14). On the surface of the unmodified PIM-1 membrane (Figure 15a), a large number of “pores” are visible, which are evenly distributed over the membrane surface. The same effect (the presence of holes onto the surface) for the pristine PIM membrane was observed in [74]. Additionally, Halder et al. [64] found the same for blend PIM1/ionic liquid-4 membranes—the formation of ellipsoidal domains. It may be associated with phase separation in the polymer matrix during preparation and modification processes. It is also worth noting that during the preparation of the membranes, all measures (degassing of the polymer solution by ultrasonic treatment, covering with a glass lid of a Petri dish for the uniform solvent evaporation) were taken to limit the possibility of pore holes in the membrane selective layer. Different crystal structures of MIL-125 and MIL-140A (Supplementary Figure S1) lead to different changes, not only in the internal morphology of the selective layer but also in the surface. The introduction of MOFs leads to a change in “pores”: with the introduction of MIL-125 (Figure 15b), a significant decrease in the number of “pores” and an increase in their sizes are observed; with the introduction of MIL-140A, the size of the “pores” does not change, but their number decreases compared to PIM-1 membranes. Comparing modified membranes, the pore sizes for PIM-1/MIL-125 membranes are larger than those for PIM-1/MIL-140A membranes. Additionally, on the surface of the modified membranes (Figure 15b,c), there are irregularities indicating the presence of MOFs, which are more expressed for the PIM-1/MIL-140A membrane. The increase in the size of the “pores” in the case of MIL-125 occurs due to the embedding of the MIL-125 in the “pore” of the PIM-1 due to its suitable size and shape (Supplementary Figure S1). This may be due to the shape and smaller size of the MIL-125 nanoparticles compared to MIL-140A (Supplementary Figure S1a), which are easily incorporated into the pores of PIM-1, causing the increase in its size due to the bulk structure of the particles. The introduction of MIL-140A does not lead to a change in the pore size but leads to a change in the microgeometry of the membrane surface (Figure 15c).

#### 3.2.3. Study by Atomic Force Microscopy (AFM)

**Figure 16:**
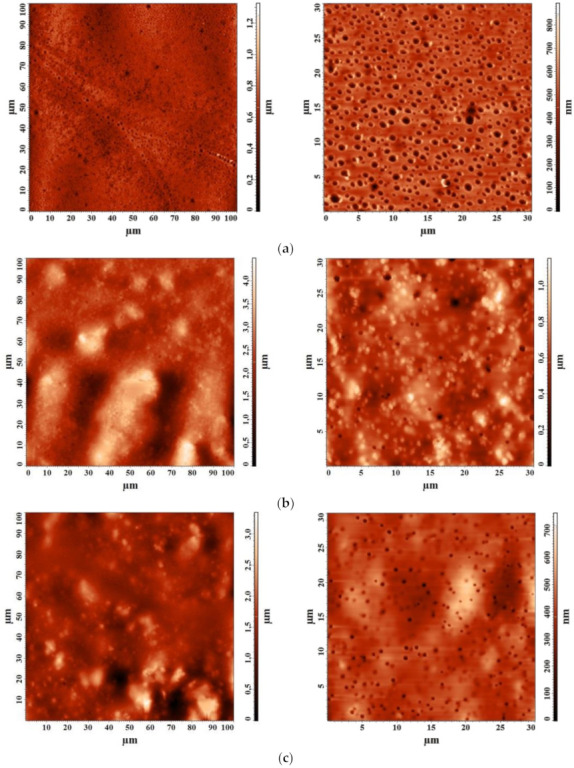
AFM images with a scanning scale of 100 × 100 and 30 × 30 μm for nanofiltration-supported (**a**) PIM-1, (**b**) PIM-1/MIL-125, and (**c**) PIM-1/MIL-140A membranes.

The introduction of MOFs into the polymer matrix changes not only the free volume of the selective layer of nanofiltration membranes but also its surface. AFM images with a scanning scale of 100 × 100 and 30 × 30 μm for nanofiltration-supported PIM-1 and PIM-1/MOFs membranes are shown in Figure 16.

The surface roughness characteristics of nanofiltration-supported PIM-1 and PIM-1/MOFs membranes were calculated based on AFM images (Figure 16). The root mean square (Rq) and average (Ra) surface roughness values are shown in Table 1.

The data presented in Table 1 demonstrate that the introduction of MOFs into the PIM-1 matrix leads to an increase in surface roughness: the introduction of MIL-125 leads to an increase in the average and root mean square roughness of ~18%, while the introduction of MIL-140A increases the average roughness of ~88% and the root mean square roughness of 99% compared to the PIM-1 membrane. Thus, the introduction of MIL-125 and the incorporation of particles into the pores slightly increases the surface roughness (also confirmed by SEM, Figure 15b), while the introduction of MIL-140A led to a change in the microgeometry of the membrane surface (Figure 15c) and a significant increase in roughness. An increase in the roughness of the membrane surface formed a large effective area in contact with the separated mixture, and a larger number of sorption centers on the membrane surface, which was reflected in the transport characteristics, in particular, led to an increase in membrane permeability.

#### 3.2.4. Contact Angle Measurement

Changes in the surface properties of nanofiltration-supported PIM-1 and PIM-1/MOFs membranes were studied by measuring the contact angle of water from the side of the selective layer at ambient temperature and are presented in Table 2.

The data presented in Table 2 demonstrate that the introduction of modifiers does not lead to a significant change in the hydrophilic–hydrophobic balance of the membrane surface: the introduction of MIL-125 practically does not change the contact angle of water in comparison with the PIM-1 membrane, since MIL-125 is embedded into the “pores” and slightly changes the surface roughness, while the introduction of another modifier into the PIM-1 matrix—MIL-140A—leads to a decrease in the contact angle from 72 to 68 °C due to a change in the microgeometry of the membrane surface (Figure 15c) and an increase in roughness (Table 1).

## 4. Conclusions

Novel nanofiltration-supported membranes with improved transport characteristics were developed by the deposition of the selective layer based on a polymer of intrinsic microporosity PIM-1, modified with metal-organic frameworks MIL-125 and MIL-140A (20 wt.%), on porous substrate UPM-20. The study of modified membranes by various methods (IR, SEM, AFM, contact angle measurements) showed that the introduction of MIL-125 and MIL-140A led to a change in the structural and physicochemical properties of PIM-1/MIL-125 and PIM-1/MIL-140A membranes: a change in the size and number of “pores” was noted, as well as an increase in surface roughness of membranes.

The study of PIM-1 and PIM-1/MOFs membranes in the nanofiltration of pure solvent water and ethanol was carried out. It was demonstrated that the introduction of MIL-125 led to an increase in the membrane water permeability of ~21% and to an increase in the membrane ethanol permeability of ~28%, and the introduction of MIL-140A led to an increase in the membrane water permeability of ~73% and to an increase in the membrane ethanol permeability of ~80% compared to the unmodified PIM-1 membrane.

The investigation of PIM-1 and PIM-1/MOFs membranes in the nanofiltration of model solutions containing copper ions (Cu^2+^), mixture of heavy metal ions (Cd^2+^, Pb^2+^, Cu^2+^) in water, and anionic food dyes (alfazurin (E133) and yellow “sunset” (E110)) in ethanol demonstrated that the introduction of MOFs led to an increase in the membrane permeability, but a slight decrease in the rejection coefficient of dyes and heavy metal ions.

In the nanofiltration of real solutions containing a mixture of heavy metal ions ((Cd^2+^, Cr^3+^, Cu^2+^, Fe^3+^, Ni^2+^, Zn^2+^) in untreated wastewater and anionic food dyes (E133, E110, and E129) in dissolved caramel in a mixture of water/ethanol (50/50 vol.%) by PIM-1 and PIM-1/MOFs membranes, it was found that the introduction of MOFs led to an increase in the membrane permeability, but a slight decrease in the rejection coefficient of dyes and heavy metal ions.

It should be noted that the membranes have potential for the removal and concentration of the food dyes and heavy metal ions from model and real mixtures.

## Figures and Tables

**Table 1:** Average (Ra) and root mean square (Rq) roughness values of the surface for nanofiltration-supported PIM-1 and PIM-1/MOFs membranes.

Membrane	Ra, nm	Rq, nm
PIM-1	74.0	97.1
PIM-1/MIL-125	87.5	114.2
PIM-1/MIL-140A	139.1	193.2

**Table 2:** Contact angle of water for nanofiltration-supported PIM-1 and PIM-1/MOFs membranes.

Membrane	Contact Angle of Water, °
PIM-1	72
PIM-1/MIL-125	73
PIM-1/MIL-140A	68

## Supplementary Materials

The following are available online at https://www.mdpi.com/article/10.3390/membranes12010014/s1, Figure S1: SEM micrographs of (a) MIL-125 and (b) MIL-140A; Figure S2: FTIR of PIM-1/UPM-20 membrane and powder of MIL-125 and MIL-140A; Figure S3: XRPD of (a) MIL-125 and (b) MIL-140A.
