# Supplementary material for: Novel Mixed Matrix Membranes Based on Polymer of Intrinsic Microporosity PIM-1 Modified with Metal-Organic Frameworks for Removal of Heavy Metal Ions and Food Dyes by Nanofiltration
Source: Membranes (Basel). 2021 Dec 23;12(1):14. doi: 10.3390/membranes12010014 (PMC8782022; doi:10.3390/membranes12010014)
Supplement: Supplementary file 1 [file membranes-12-00014-s001.zip › membranes-1492919-supplementary.pdf]

Novel mixed matrix membranes based on polymer of intrinsic microporosity PIM-1 modified with metal-organic frameworks for removal of heavy metal ions and food dyes by nanofiltration

Anna Kuzminova [1,*], Mariia Dmitrenko[1], Andrey Zolotarev[1], Daria Poloneeva[1], Artem Selyutin[1], Alexei Emeline[1], Alexey Yushkin[2], Andrew Foster[3], Peter Budd[3] and Sergey Ermakov[1]

[1] St. Petersburg State University, 7/9 Universitetskaya nab., St. Petersburg 199034, Russia

[2] A.V.Topchiev Institute of Petrochemical Synthesis RAS, 29 Leninsky prospekt, 119991, Moscow, Russian Federation

[3] University of Manchester, Department of Chemistry, Oxford Road, Manchester M13 9PL, United Kingdom

S1. Synthesis and characterization of MOFs particles

Materials

$C_8H_6O_4$ (1,4-benzdicarboxylic acid, BDC, Sigma-Aldrich), $ZrCl_4$ (Zirconium(IV) chloride, Sigma-Aldrich), $TiO_4C_{12}H_{28}$ (Titanium(IV) isopropoxide, Sigma Aldrich, 97%), $C_2H_4O_2$ (Acetic acid anhydrous, AcOH, 99.999%), C_3H_7NO (N,N-Dimethylformamide, DMF, Vekton), CH_3OH (Methanol, MeOH, Vekton). Starting materials and solvents were used without further purification.

MIL-140A

MIL-140A was synthesized by the following procedure: 6.4 mmol of 1,4-benzdicarboxylic acid and 6.4 mmol of zirconium chloride were dissolved in 100 mL of DMF, then 1.5 mL of glacial acetic acid was added. The obtained solution was placed in a Teflon autoclave and heated up to 150 °C. The reaction mixture was withstood for 24 h under this temperature. Then the mixture was cooled down to room temperature and the obtained precipitate was filtered and washed with DMF three times. After it, the powder was dried at 100°C under vacuum, then immersed in MeOH for 20 min with following centrifugation. The last step was repeated two times more. The resulted product was dried at 80 °C under vacuum conditions.

MIL-125

MIL-125 was synthesized following procedure [1] with a simplification of the whole process. Shortly, 21.2 mmol of 1,4-benzdicarboxylic acid was dissolved in a mixture of DMF (56 mL) and methanol (14 mL). After full dissolution of the linker, 4.2 mL of titanium isopropoxide was added

to the solution. The obtained mixture was transferred into a Teflon autoclave and was kept at 110 °C for 72 h. After cooling down the system to room temperature, the obtained product was filtered and washed with DMF, then methanol. The obtained powder was dried under at 70 °C in the air, and then at 100 °C under vacuum.

Scanning electron microscopy

Figure S1 shows SEM micrographs of MIL-125 and MIL-140A.

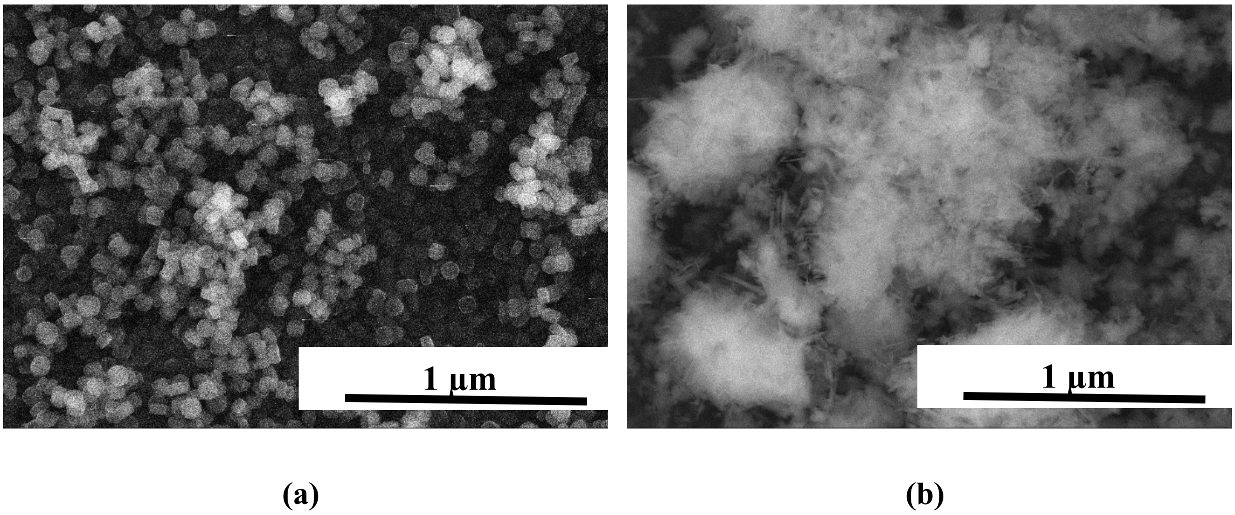

Figure S1. SEM micrographs of (a) MIL-125 and (b) MIL-140A.

The micrographs show that the sizes and shapes of MIL-125 and MIL-140A correspond to the crystal structure and are very different for the two samples. MIL-125 crystals are parallelepipeds with rounded corners and edges (Figure S1a) 600*500*400 nm in size. MIL-140A crystals are flat needles no less than 5 µm long and no more than 100 nm thick (Figure S1b). Different crystal structures of MIL-125 and MIL-140A lead to different changes not only in the internal morphology of the selective layer, but also in the surface.

Low-temperature nitrogen adsorption (BET)

Physisorption measurements with nitrogen were performed at 77K with ASAP 2020 MP analyzer (Micromeritics, USA). The resulting BET surface area is around 493.4±0.2 m^2 per gram for MIL-140A and 1565±2.3 m^2 per gram for MIL-125. The calculation of the pore volume was carried out according to the Horvath-Kawazoe method. Maximum pore volume at p/p° = 0.150 for MIL-140A was calculated as 0.189 cm^3/g and median pore width was 0.87 nm. For MIL-125 it was 0.602cm^3/g at p/p° = 0.152. Median pore width for MIL-125 was 1.12 nm.

Infrared spectroscopy

The BRUKER-TENSOR 27 spectrometer was used to determine the structure of PIM-1/UPM-20 and MOFs powder (MIL-125 and MIL-140A). IR spectroscopy was performed in the

range 600-4000 cm^{-1} at 25°C. The measurement of the PIM-1/UPM-20 was carried out from the side of the selective layer of the supported membrane with an attenuated total reflectance (ATR) accessory (PIKE Technologies, Moscow, Russia). The measurement of MOFs was carried out in KBr by with an attenuated total reflectance (ATR) accessory. FTIR of PIM-1/UPM-20, MIL-125 and MIL-140A are presented in Figure S2.

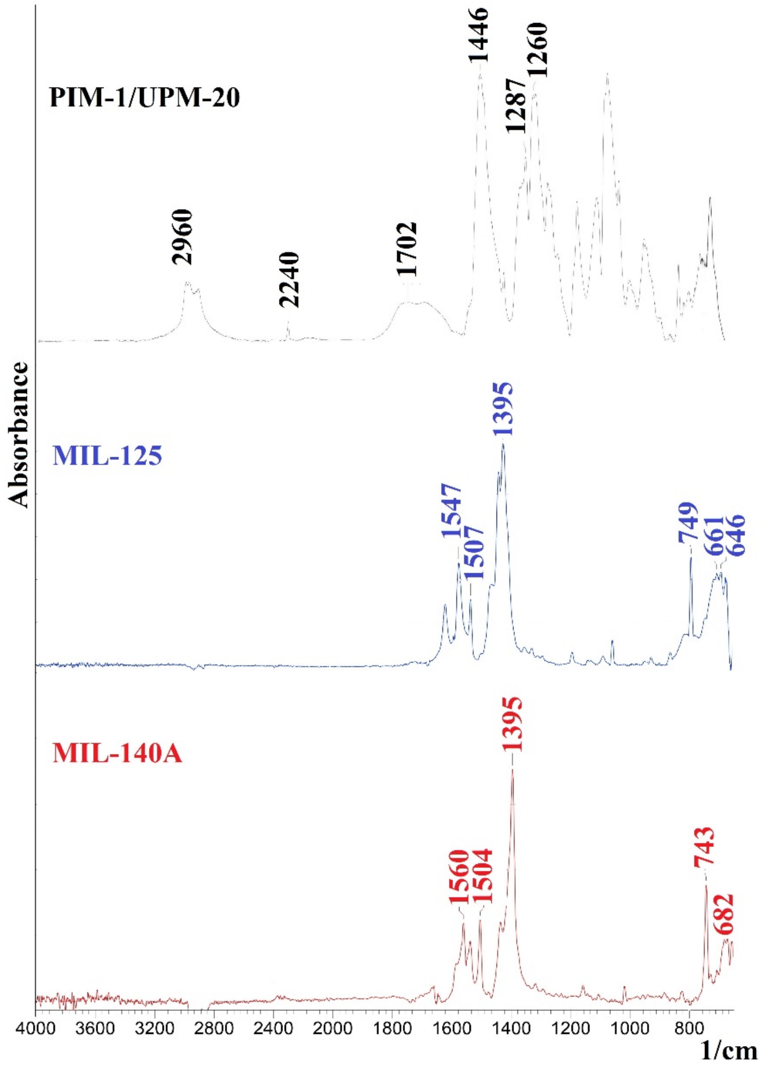

Figure S2. FTIR of PIM-1/UPM-20 membrane and powder of MIL-125 and MIL-140A.

The characteristic peaks are presented in the IR spectra of PIM-1. The peak at 2240 cm^{-1} indicates the stretching of the nitrile group, the peak at 2800-2900 cm^{-1} corresponds to –C–H [2], the peak at 1702 cm^{-1} corresponds to C=N [3], the peak at 1446 cm^{-1} corresponds to –CH bonds from the C-CH_3 and -CH_2 groups [4], the group of peaks at 1250-1350 cm^{-1} corresponds to the stretching of the -C-O- bond [2].

MIL-125 spectrum exhibits two intense peaks of asymmetric (with maximum at 1547 cm^{-1}) and symmetric (with maximum at 1395 cm^{-1}) vibrations of carboxylate groups of 1,4-benzdicarboxylic acid (BDC) linker. A narrow minor peak at 1507 cm^{-1} might be due to C=C

stretching of the benzene ring in the linker [5]. Peaks below 800 cm^{-1} belong to Ti-O vibration modes [6].

In general, spectrum of MIL-140A is quite similar MIL-125, which is due to identical linkers. It also exhibits two peaks of asymmetric (with maximum at 1560 cm^{-1}) and symmetric (maximum at 1395 cm^{-1}) vibrations of carboxylate groups of the linker. Similarly, to MIL-125 peak at 1504 cm^{-1} might be due to C-C stretching in the benzene ring of BDC. Peaks below 800 cm^{-1} might be coming from Zr-O vibrations [5].

X-Ray powder diffraction (XRPD)

X-Ray powder diffraction (XRPD) analysis was performed on Rigaku «MiniFlexII» powder diffractometer (CuKα-radiation, $\lambda CuK_{\alpha 1}$ = 1.54051 Å и $\lambda CuK_{\alpha 2}$ = 1.54443 Å) of the anode copper in the range of scan angles 2θ from 0 to 60°. Identification of the qualitative composition was carried out with the help of using diffraction data «Powder Diffraction File». XRPD of MIL-125 and MIL-140A are presented in Figure S3.

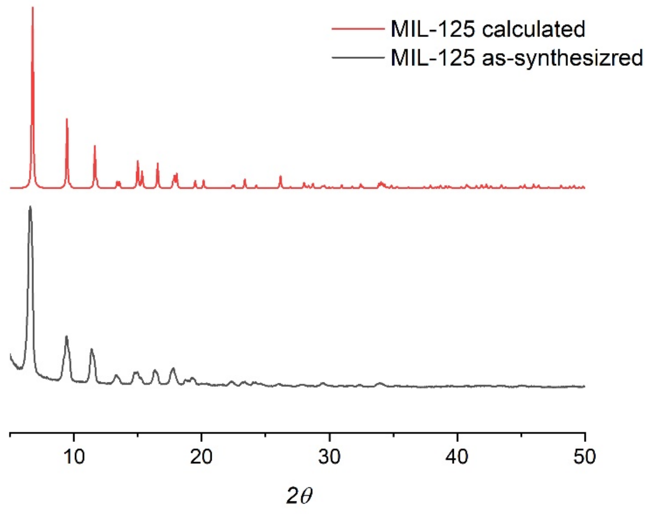

(a)

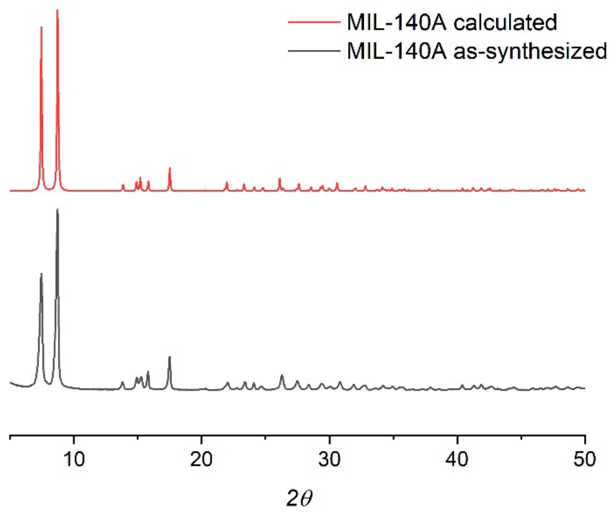

(b)

Figure S3. XRPD of (a) MIL-125 and (b) MIL-140A.

The powder XRD patterns (Figure S3) of the obtained MOFs are crystaline and correspond to calculated patterns of the investigated MOFs [7,8].

1. Santaclara, J.G.; Nasalevich, M.A.; Castellanos, S.; Evers, W.H.; Spoor, F.C.M.; Rock, K.; Siebbeles, L.D.A.; Kapteijn, F.; Grozema, F.; Houtepen, A.; et al. Organic Linker Defines the Excited-State Decay of Photocatalytic MIL-125(Ti)-Type Materials. *ChemSusChem* **2016**, *9*, 388–395, doi:10.1002/cssc.201501353.
2. Yahia, M.; Phan Le, Q.N.; Ismail, N.; Essalhi, M.; Sundman, O.; Rahimpour, A.; Dal-Cin, M.M.; Tavajohi, N. Effect of incorporating different ZIF-8 crystal sizes in the polymer of intrinsic microporosity, PIM-1, for CO2/CH4 separation. *Microporous Mesoporous Mater.* **2021**, *312*, 110761, doi:10.1016/j.micromeso.2020.110761.
3. Satilmis, B. Amidoxime Modified Polymers of Intrinsic Microporosity (PIM-1); A Versatile Adsorbent for Efficient Removal of Charged Dyes; Equilibrium, Kinetic and Thermodynamic Studies. *J. Polym. Environ.* **2020**, *28*, 995–1009, doi:10.1007/s10924-020-01664-4.
4. Chen, W.; Zhang, Z.; Hou, L.; Yang, C.; Shen, H.; Yang, K.; Wang, Z. Metal-organic framework MOF-801/PIM-1 mixed-matrix membranes for enhanced CO2/N2 separation performance. *Sep. Purif. Technol.* **2020**, *250*, 117198, doi:10.1016/j.seppur.2020.117198.
5. Kuzminova, A.I.; Dmitrenko, M.E.; Poloneeva, D.Y.; Selyutin, A.A.; Mazur, A.S.; Emeline, A. V.; Mikhailovskii, V.Y.; Solovyev, N.D.; Ermakov, S.S.; Penkova, A. V. Sustainable composite pervaporation membranes based on sodium alginate modified by metal organic frameworks for dehydration of isopropanol. *J. Memb. Sci.* **2021**, *626*, 119194, doi:10.1016/j.memsci.2021.119194.
6. Han, L.; Zhang, X.; Wu, D. Construction and characterization of BiOI/NH2-MIL-125(Ti) heterostructures with excellent visible-light photocatalytic activity. *J. Mater. Sci. Mater. Electron.* **2019**, *30*, 3773–3781, doi:10.1007/s10854-018-00660-8.
7. Smalley, A.P.; Reid, D.G.; Tan, J.C.; Lloyd, G.O. Alternative synthetic methodology for amide formation in the post-synthetic modification of Ti-MIL125-NH2. *CrystEngComm* **2013**, *15*, 9368, doi:10.1039/c3ce41332b.
8. D'Amato, R.; Donnadio, A.; Carta, M.; Sangregorio, C.; Tiana, D.; Vivani, R.; Taddei, M.; Costantino, F. Water-Based Synthesis and Enhanced CO 2 Capture Performance of Perfluorinated Cerium-Based Metal–Organic Frameworks with UiO-66 and MIL-140 Topology. *ACS Sustain. Chem. Eng.* **2019**, *7*, 394–402, doi:10.1021/acssuschemeng.8b03765.
